# Lenticular nucleus correlates of general self-efficacy in young adults

**DOI:** 10.1007/s00429-017-1406-2

**Published:** 2017-03-28

**Authors:** Seishu Nakagawa, Hikaru Takeuchi, Yasuyuki Taki, Rui Nouchi, Yuka Kotozaki, Takamitsu Shinada, Tsukasa Maruyama, Atsushi Sekiguchi, Kunio Iizuka, Ryoichi Yokoyama, Yuki Yamamoto, Sugiko Hanawa, Tsuyoshi Araki, Carlos Makoto Miyauchi, Daniele Magistro, Kohei Sakaki, Hyeonjeong Jeong, Yukako Sasaki, Ryuta Kawashima

**Affiliations:** 1 Division of Psychiatry, Tohoku Medical and Pharmaceutical University, Sendai, Japan; 2 Department of Human Brain Science, Institute of Development, Ageing and Cancer, Tohoku University, 4-1 Seiryo-machi, Aoba-ku, Sendai, 980-8575 Japan; 3 Division of Developmental Cognitive Neuroscience, Institute of Development, Ageing and Cancer, Tohoku University, Sendai, Japan; 4 Division of Medical Neuroimaging Analysis, Department of Community Medical Supports, Tohoku Medical Megabank Organization, Tohoku University, Sendai, Japan; 5 Department of Nuclear Medicine and Radiology, Institute of Development, Ageing and Cancer, Tohoku University, Sendai, Japan; 6 Creative Interdisciplinary Research Division, Frontier Research Institute for Interdisciplinary Science (FRIS), Tohoku University, Sendai, Japan; 7 Smart Ageing International Research Center, Institute of Development, Ageing and Cancer, Tohoku University, Sendai, Japan; 8 Department of Adult Mental Health, National Institute of Mental Health, National Center of Neurology and Psychiatry, Kodaira, Tokyo Japan; 9 Department of Psychiatry, Tohoku University Graduate School of Medicine, Sendai, Japan; 10 School of Medicine, Kobe University, Kobe, Japan; 11 Japan Society for the Promotion of Science, Tokyo, Japan; 12 Advantage Risk Management Co., Ltd, Tokyo, Japan; 13 Graduate School of Arts and Sciences, The University of Tokyo, Tokyo, Japan; 14 National Centre for Sport and Exercise Medicine (NCSEM), Loughborough University, Leicester, UK; 15 The NIHR Leicester-Loughborough Diet, Lifestyle and Physical Activity Biomedical Research Unit, Leicester, UK; 16 School of Sport, Exercise, and Health Sciences, Loughborough University, Leicester, UK; 17 Advanced Brain Science, Institute of Development, Aging and Cancer, Tohoku University, Sendai, Japan

**Keywords:** General self-efficacy, Mean diffusivity, Pallidus, Putamen

## Abstract

General self-efficacy (GSE) is an important factor in education, social participation, and medical treatment. However, the only study that has investigated the direct association between GSE and a neural correlate did not identify specific brain regions, rather only assessed brain structures, and included older adult subjects. GSE is related to motivation, physical activity, learning, the willingness to initiate behaviour and expend effort, and adjustment. Thus, it was hypothesized in the present study that the neural correlates of GSE might be related to changes in the basal ganglia, which is a region related to the abovementioned self-efficacy factors. This study aimed to identify the brain structures associated with GSE in healthy young adults (*n* = 1204, 691 males and 513 females, age 20.7 ± 1.8 years) using regional grey matter density and volume (rGMD and rGMV), fractional anisotropy (FA) and mean diffusivity (MD) analyses of magnetic resonance imaging (MRI) data. The findings showed that scores on the GSE Scale (GSES) were associated with a lower MD value in regions from the right putamen to the globus pallidum; however, there were no significant association between GSES scores and regional brain structures using the other analyses (rGMD, rGMV, and FA). Thus, the present findings indicated that the lenticular nucleus is a neural correlate of GSE.

**Electronic supplementary material:**

The online version of this article (doi:10.1007/s00429-017-1406-2) contains supplementary material, which is available to authorized users.

## Introduction

Self-efficacy beliefs manifest through cognitive, motivational, affective, and selection processes to determine how people feel, think, motivate themselves, and behave (Bandura 1977). Self-efficacy in students is a highly effective predictor of motivation and learning, because it is sensitive to subtle changes in performance context, interacts with self-regulated learning processes, and mediates academic achievements (Zimmerman 2000). Increasing self-efficacy is also an effective method with which to increase physical activity (Ashford et al. 2010; Bauman et al. 2012).

General self-efficacy (GSE) has been defined as one’s perception of their own ability to perform in a variety of different situations (Judge et al. 1998), and it reflects a generalization across various domains of functioning in which people judge how efficaciously they cope with a broad range of stressful or challenging demands (Luszczynska et al. 2005). GSE also refers to one’s confidence in their general capacity to handle tasks (Suzuki et al. 2011) and to generalize their behaviour towards a stimulus other than the target stimulus (Sherer et al. 1982). That is, GSE is a situation-independent belief in one’s competence (Scherbaum et al. 2006). Importantly, the willingness to initiate behaviour and expend effort while completing tasks, being persistent in the face of adversity, and enhanced personal adjustment are primary components of GSE (Sherer et al. 1982). For example, stress management training for university students enhances GSE and reduces anxiety (Molla Jafar et al. 2015), and GSE is related to lower levels of post-traumatic stress reactions during the first months after a disaster (Nygaard et al. 2016). A population-based cross-sectional study investigating GSE found that it is an important factor to consider in the relationship between personality and perceived stress (Ebstrup et al. 2011). Thus, GSE seems to be an important factor during the adjustment of one’s behaviour.

A previous study investigating brain structures that may be directly related to GSE demonstrated that scores on the GSE Scale (GSES) are positively associated with total brain volume and total grey matter volume in healthy senior women between 65 and 75 years of age (Davis et al. 2012). However, in a previous study from our research group that utilized regional white matter density (rWMD) and fractional anisotropy (FA) to assess young adults, no regions were significantly or directly correlated with the GSES (Nakagawa et al. 2015). The sample size we reported previously (*N* = 776) was smaller than that in this study (*N* = 1204). To the best of our knowledge, no structural brain studies have successfully identified significant, specific, direct anatomical correlates of GSE in healthy young adults. Furthermore, previous brain imaging studies related to GSE in healthy individuals have predominantly focused on the relationship between particular brain regions and the factors related to self-efficacy, i.e., motivation, physical activity (Nakagawa et al. 2016), learning (van der Meer and Redish 2011; Arsalidou et al. 2013), willingness to initiate behaviour and expend effort (van der Meer and Redish 2011), and adjustment (Leisman et al. 2014), rather than GSE itself.

One structural brain study that investigated the factors related to GSE in young adults showed that the degree of motivation is associated with higher mean diffusivity (MD) values in the right putamen, globus pallidum, and caudate, and that the degree of physical activity is associated with the right putamen (Nakagawa et al. 2016). MD, which is another measure of diffusion tensor imaging (DTI), uses the rate of diffusivity and a direction-independent measure of average diffusivity that reflects water motility in a voxel (Acosta-Cabronero et al. 2010). As summarised in a previous study from our research group (Nakagawa et al. 2016), there are three diffusivities: the diffusion coefficient along the direction of maximal diffusion (axial diffusivity *λ*1) and two diffusion coefficients along the two orthogonal directions embedded in the plane perpendicular to the main diffusion direction (*λ*2 and *λ*3) (Acosta-Cabronero et al. 2010). The average diffusivity of *λ*1, *λ*2, and *λ*3 is known as MD and can be inferred from the overall dimensions of the diffusion ellipsoid (Acosta-Cabronero et al. 2010). Reductions in MD are considered to reflect changes in tissue, such as astrocyte swelling, synaptic changes, dendritic spine changes, and angiogenesis, that are caused by neural plasticity (Sagi et al. 2012; Johansen-Berg et al. 2012). As we mentioned previously (Takeuchi et al. 2016a), MD is considered to represent axonal and myelin integrity, including of capillaries, synapses, spines, and macromolecular proteins; properties of myelin, membranes, and axons; the shape of neurons or glia; enhanced tissue organisation. On the other hand, FA is not only related to axonal count and density and degree of myelination, but also to fibre organisation (degree of parallel organisation of axons) (Winston 2012). Therefore, MD and FA measure different microstructural brain properties (Takeuchi et al. 2016a).

It has also been shown that reinforcement learning, category learning, sequential decision-making, and learning based on the evaluation of outcomes are related to the basal ganglia (van der Meer and Redish 2011; Arsalidou et al. 2013), and that the willingness to initiate behaviour and expend effort is associated with the ventral stratum (van der Meer and Redish 2011). The basal ganglia, which regulates motor control, is also an important region for adaptive functioning (Leisman et al. 2014). Regional gray matter density (rGMD) and volume (rGMV) may identify significant effects in different regions (Mechelli et al. 2005). Based on these findings, the present study hypothesised that the degree of self-efficacy is related to the basal ganglia, and that this relationship can be demonstrated using rGMD, rGMV, FA, and MD analyses.

Thus, the present study utilized voxel-based morphometry (VBM) to analyse rGMD, rGMV FA, and MD results to identify the neural correlates of GSE. The purpose of the present study was to identify the anatomical correlates of GSE in young people using rGMD, rGMV, FA, and MD analyses. Importantly, adjustment reaction due to social and academic stress in school is common in individuals of the age represented by this sample. Moreover, social cognitive theory states that state anxiety affects self-efficacy measures (Bandura 1988). Accordingly, we determined whether there was any correlation between the neural correlates of GSE and state anxiety.

## Methods

### Subjects

The present study included 1204 healthy right-handed individuals (691 males and 513 females) with a mean age of 20.7 ± 1.8 years. Written informed consent was obtained from each participant prior to beginning the study, all study procedures were approved by the Ethics Committee of Tohoku University, and all experiments were performed in accordance with the approved guidelines. For more details regarding the study procedures, please see Supplemental Methods.

### Psychological outcome measures

#### Assessment of general self-efficacy (GSE)

The GSES was developed by Sherer et al. (1982), and Sherer and Adams (1983) to measure GSE for events that occur in various everyday settings. The validity of the GSES for the variables evaluated in the present study is consistent across countries and participants; thus, this psychometric scale appears to tap into a universal construct and yields meaningful relationships with other psychological constructs (Luszczynska et al. 2005). The total score on the Japanese version of the GSES, developed by Sherer et al. (1982), has satisfactory test–retest reliability, internal consistency, and validity for all generations regardless of sex (Narita et al. 1995). This scale includes a questionnaire with 23 items, each rated on a 5-point Likert scale that results in a total score ranging from 23 to 115, where higher scores indicate a greater sense of GSE (10 reverse scores are included). The scale items focus on the following areas: willingness to initiate behaviour and expend effort when completing a behaviour and persistence in the face of adversity (Sherer et al. 1982). Examples of the questions include “If I can’t do a job the first time, I keep trying until I can” and “Failure just makes me try harder”.

#### Assessment of state anxiety

In the present study, anxiety was assessed using the state anxiety subscale of the Japanese version of the State–Trait Anxiety Inventory (STAI; Nakazato and Mizuguchi 1982; Spielberger et al. 1983). The state anxiety scale evaluates the current state of anxiety, asking how respondents feel ‘right now’ using items that measure subjective feelings of apprehension, tension, nervousness, worry, and activation/arousal of the autonomic nervous system. In total, 20 state anxiety items are rated on 4-point scales, as follows: 1 (not at all), 2 (somewhat), 3 (moderately so), and 4 (very much so) (Spielberger et al. 1983).

#### Psychometric measures of general intelligence

The Raven’s Advanced Progressive Matrix (RAPM), which is a widely used measure of general intelligence (Raven 1998), was utilized in the present study. This measure was adjusted to examine the effects of general intelligence on brain structures (Haier et al. 2004; Colom et al. 2006; Narr et al. 2007) to exclude the possibility that a significant correlation between MD and the GSES score was due to either an association between the GSES score and general intelligence or an association between MD and general intelligence.

### Behavioural data analyses

All behavioural data were analysed using the IBM SPSS Statistics 22.0 software package (IBM Corp.; Armonk, NY, USA). Differences between males and females in terms of age and scores on the cognitive measures (RAPM, state anxiety, and GSES) were analysed by one-way analysis of variance (ANOVA); a *P* value < 0.05 was considered to indicate statistical significance.

### Image acquisition

#### Structural MRI

All magnetic resonance imaging (MRI) data were acquired using a 3 T Philips Achieva scanner (Philips Medical Systems, Best, Netherlands). Three-dimensional high-resolution T1-weighted images were collected using a magnetisation-prepared rapid gradient-echo sequence with the following parameters: 240 × 240 matrix, TR = 6.5 ms, TE = 3 ms, TI = 711 ms, FOV = 24 cm, 162 slices, in plane resolution = 1.0 × 1.0 mm, slice thickness = 1.0 mm, and a scan duration of 483 s.

Diffusion-weighted data were acquired using a spin-echo echo-planar imaging (EPI) sequence with the following parameters: TR = 10,293 ms, TE = 55 ms, FOV = 22.4 cm, 2 × 2 × 2 mm^3^ voxels, 60 slices, SENSE reduction factor = 2, and number of acquisitions = 1. The diffusion weighting was isotropically distributed along 32 directions (b value = 1000 s/mm^2^), and three images with no diffusion weighting (*b* value = 0 s/mm^2^; *b* = 0 images) were acquired using the spin-echo EPI sequence (TR = 10,293 ms, TE = 55 ms, FOV = 22.4 cm, 2 × 2 × 2 mm^3^ voxels, 60 slices). Acquisitions for phase correction and signal stabilization were performed, but these data were not used as part of the reconstructed images. For more details regarding these procedures, please see Supplemental Methods. The descriptions in this subsection were reproduced mostly from a previous study that employed similar methods (Takeuchi et al. 2016a).

### Pre-processing and analyses of structural data

#### VBM data

All pre-processing of the MRI data was performed using Statistical Parametric Mapping software (SPM12; Wellcome Department of Cognitive Neurology, London, UK) according to the protocol described for VBM analyses in a previous report from our research group (Hashimoto et al. 2015). First, the rGMD values were calculated, and then the diffeomorphic anatomical registration was performed using the diffeomorphic anatomical registration exponentiated lie algebra (DARTEL) process implemented in SPM12. In this process, the DARTEL-imported images of grey and white matter tissue probability maps were used to create the abovementioned segmentation process. All images were smoothed by convolving them using an isotropic Gaussian kernel of 8 mm full-width at half maximum (FWHM). For additional details, please see Supplemental Methods.

#### FA and MD data

All pre-processing and analyses of the imaging data were performed using SPM8 implemented in Matlab (Mathworks Inc.; Natick, MA, USA). The MD map was calculated from the collected images using a commercially available diffusion tensor analysis package (Philips Medical Systems, Best, Netherlands) on the MR console. These procedures involved corrections for motion and distortion caused by eddy currents, and all calculations were performed using a previously described method (Le Bihan et al. 2001). Briefly, the MD images of the participants were normalized using a previously validated DARTEL-based registration process to develop images with 1.5 × 1.5 × 1.5 mm^3^ voxels. Next, tissues that were least likely to be grey or white matter were carefully removed, and the images were smoothed by convolving them using an isotropic Gaussian kernel of 8 mm FWHM. For additional details, please see Supplemental Methods.

### Statistical group-level analyses of imaging and behavioural data

We did not include psychological measures related to GSE in the whole-brain multiple regression analyses, which were used to investigate the association between GSES and rGMD. We regarded state anxiety and GSES as partly overlapping, a neural basis that could not be regressed. Accordingly, as we explained previously (Takeuchi et al. 2016b), we did not regard state anxiety as a confounding variable.

#### VBM data

A whole-brain multiple regression analysis performed in SPM12 was used to assess the association between rGMD or rGMV and GSES scores. The covariates included sex, age, RAPM score, and total intracranial brain volume (TIV), which were calculated as follows: total GM volume + total WM volume + total cerebrospinal fluid (CSF) volume. For each covariate, the overall mean was used for mean centring.

Next, we investigated whether the relationship between rGMD or rGMV and GSES scores differed between males and females (i.e. whether the interaction between sex and GSES scores affected rGMD or rGMV). In the two whole-brain analyses, we used a voxel-wise analysis of covariance (ANCOVA) in which sex difference was a group factor (using the full factorial option of SPM12). Age, RAPM score, GSES score, and TIV were covariates in one analysis.

Correction for multiple comparisons was performed using threshold-free cluster enhancement (TFCE) (Smith and Nichols 2009) with randomised (5000 permutations) nonparametric testing in the TFCE toolbox (http://dbm.neuro.uni-jena.de/tfce/). A family-wise error (FWE) corrected threshold of *P* < 0.05 was applied.

#### FA and MD data

A voxel-by-voxel regression analysis was performed using the FA or MD value at each voxel as the dependent variable and age, sex, RAPM score, and GSES score as the independent variables. The analyses were limited to areas within the grey and white matter masks that were created using the procedures described above. Additionally, we investigated whether the relationship between FA or MD and GSES scores differed between males and females (i.e. whether the interaction between sex and GSES scores affected FA or MD). In the two whole-brain analyses, we used voxel-wise ANCOVA, in which sex difference was a group factor (using the full factorial option of SPM8). Age, RAPM score, GSES score, and TIV were covariates in one analysis. Correction for multiple comparisons was performed using TFCE (Smith and Nichols 2009) with randomised (5,000 permutations) nonparametric testing in the TFCE toolbox. A FWE-corrected threshold of *P* < 0.05 was applied. For additional details, please see Supplemental Methods.

#### Regions of interest (ROI) analysis of the association between MD and GSES

Subsequently, after identifying the MD correlates of GSES, we employed an ROI approach (Takeuchi et al. 2016c) to determine whether the MD correlates of the GSES were also associated with state anxiety. These areas included the right globus pallidum and putamen. Both ROIs were constructed using the WFU PickAtlas Tool (http://www.fmri.wfubmc.edu/cms/software#PickAtlas) (Maldjian et al. 2003, 2004). The mask images of the ROIs were generated using the Brodmann area option in the PickAtlas Tool. Subsequently, the mean MD values of these images were extracted from the aforementioned normalised images. We limited the areas from which these values were extracted from to those that showed ‘gray matter tissue probability + white matter tissue probability >0.999’ in the custom template mentioned above (Takeuchi et al. 2016c).

#### Associations between MD and state anxiety

Structural equation modelling (SEM) is useful for assessing mediation because it offers several interesting, alternative ways to explore a mediation effect (Preacher and Hayes 2004). Hence, we conducted SEM to demonstrate that the link between GSES and the mean MD values within the ROIs (right globus pallidum and putamen) were mediated by individual differences in state anxiety. We used linear structural equation systems (AMOS 18, SPSS, Inc., Chicago, IL; 2009) to explore the relationship between GSES and state anxiety scale scores.

We constructed a model (Model 1: increasing MD values in the right putamen and globus pallidum decreased the GSE affected by state anxiety). We constructed modified Model 1 with a correlation between the right putamen and globus pallidum (Model 2). We also constructed an inverse model (Model 3: increasing the GSE affected by state anxiety decreased the MD values in the right putamen and globus pallidum).

## Results

### Behavioural data

**Fig. 1:**
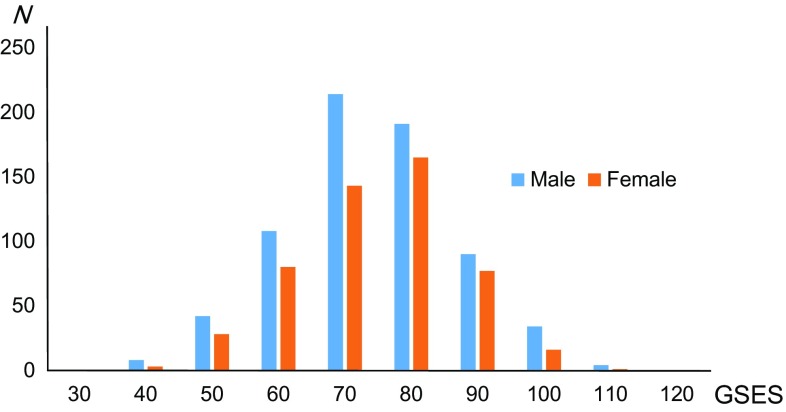
Distribution of GSES scores in males and females. Histograms showing the distribution of GSES scores in males and females. *GSES* general self-efficacy scale

Table 1 shows the means and standard deviations (SD) for age and the RAPM scores, State Anxiety scores, and GSES scores. Figure 1 depicts the distributions of the GSES scores in males and females. There were significant differences between males and females in the RAPM scores (*P* < 0.05, one-way ANOVA) but not the GSES scores (*P* = 0.609).

**Table 1:** Sex differences in age and scores on the RAPM, state anxiety, and GSES using one-way ANOVA (means ± SD)

Measure	Total	Males (*N* = 691)	Females (*N* = 513)	*P*	*F*
Age	20.7 (1.8)	20.8 (1.9)	20.5 (1.6)	0.025^*^	5.04
RAPM	28.5 (3.9)	28.8 (3.9)	28.1 (3.8)	0.003^**^	9.13
Sate anxiety	39.9 (7.8)	40.0 (7.9)	39.8 (7.7)	0.827	0.05
GSES	69.6 (12.2)	69.4 (12.3)	69.8 (11.8)	0.609	0.26

*ANOVA* analysis of variance, *GSES* General Self-Efficacy Scale, *RAPM* Raven’s Advanced Progressive Matrix, *SD* standard deviation

^*^ *P* < 0.05, ^**^ *P* < 0.01

### MRI data

#### Analysis of VBM data

After controlling for sex, age, RAPM scores, and TIV, there were no significant positive or negative correlations between GSES scores and rGMD, or rGMV at each voxel at a FWE-corrected threshold of *P* < 0.05, based on the TFCE method at the whole-brain level.

#### Analysis of FA and MD data

**Fig. 2:**
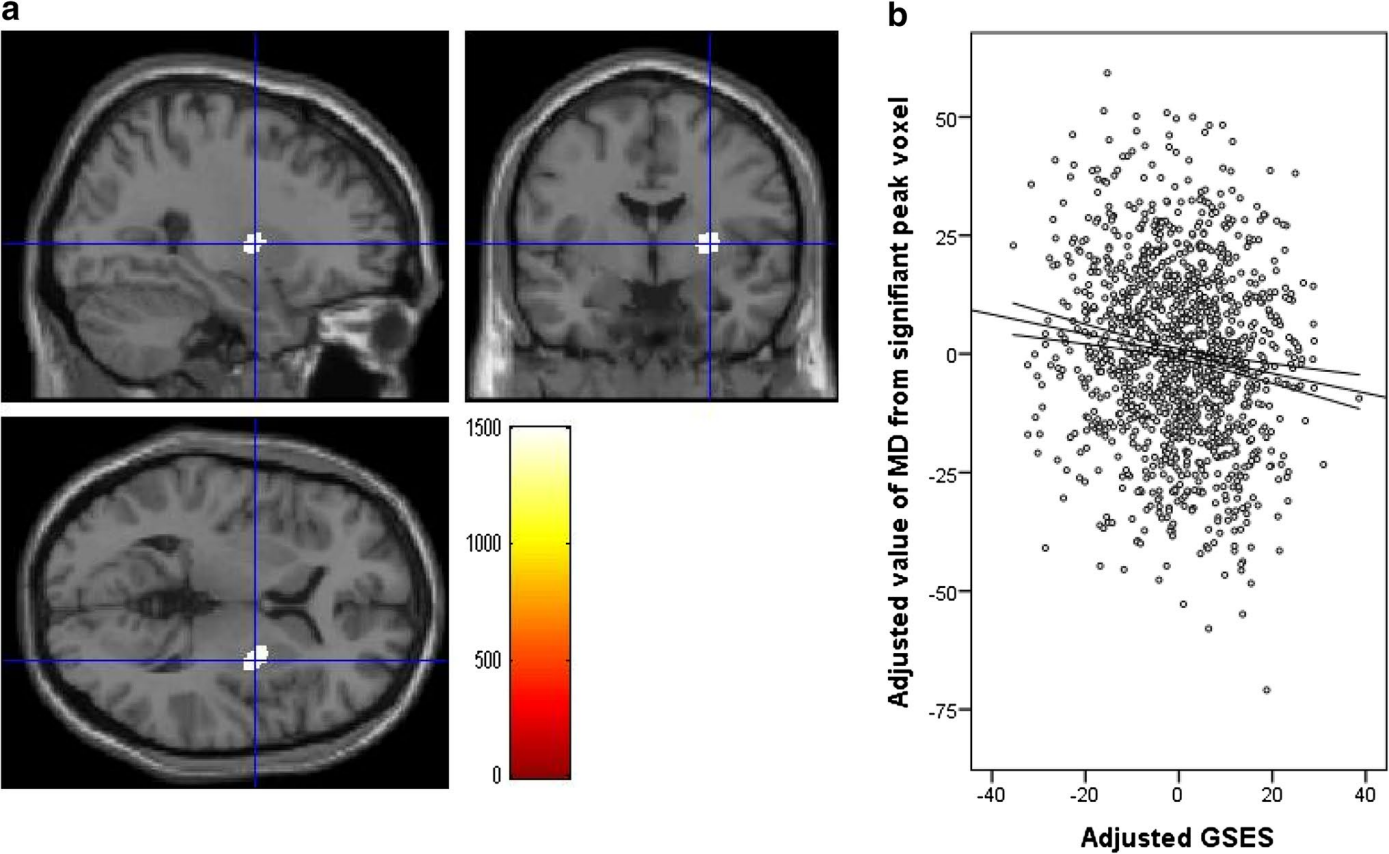
Regions correlated with MD and GSES scores. The present results were determined based on a family-wise error (FWE)-corrected threshold of *P* < 0.05 with a threshold-free cluster enhancement (TFCE) based on 5000 permutations; the results were corrected at the whole-brain level. Regions showing correlations were overlaid on a single T1 image in the SPM8 toolbox. The *red-to-yellow colour scale* indicates the strength of the TFCE value for the negative correlation between the MD and GSES scores; areas with significant correlations were identified in the right putamen and globus pallidum (**a**). Also shown are *residual plots* with *trend lines* depicting the correlations between residuals in the multiple regression analysis, which included the MD value of each significant peak voxel as a dependent variable and the GSES scores and other confounding factors as independent variables; 95% confidence intervals for the *trend lines* are shown (**b**). *GSES* general self-efficacy scale, *MD* mean diffusivity

A whole-brain multiple regression analysis that controlled for sex, age, RAPM scores, and TIV revealed a significant negative correlation between GSES scores and MD in areas corresponding to widespread regions from the right putamen to the right putamen and globus pallidum (*x, y, z* = 29, −3, 5; TFCE = 1501.51, *P* = 0.044, *k* = 206, with FWE correction) at the whole-brain level (Fig. 2). There were no positive significant correlations between GSES scores and MD at the same analysis at the whole-brain level.

We found no significant correlations between GSES scores and FA using the same analyses abovementioned.

#### Interaction effects of sex and GSES on brain structures

Using data from both sexes with respect to the covariates of age, RAPM, TIV, and GSES score, ANCOVA revealed no significant effect of the interaction between GSES scores and sex on rGMD, rGMV, FA, or MD using the TFCE method with FWE corrected to *P* < 0.05 at the whole-brain level.

#### Associations between MD and state anxiety

**Fig. 3:**
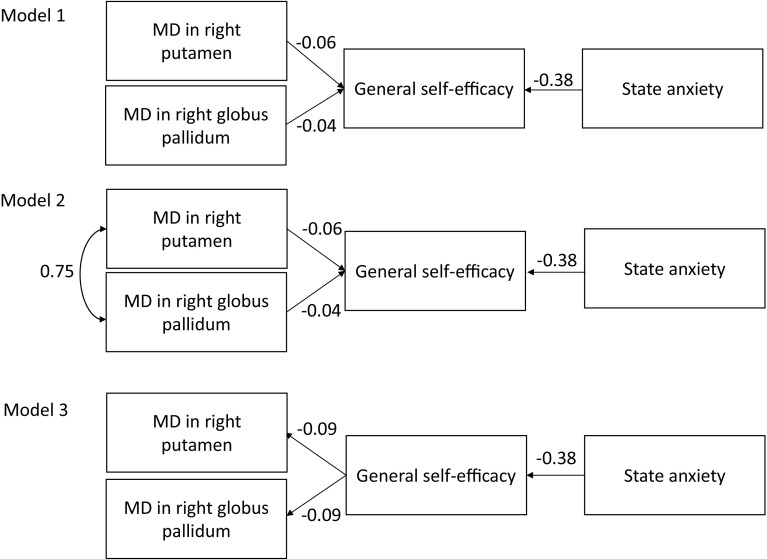
*Model 1* increased MD values in the lenticular nucleus reduced the GSE affected by state anxiety. *Model 2* increased MD values in the lenticular nucleus reduced the GSE affected by state anxiety, with a correlation between the globus pallidum and putamen. *Model 3* GSE affected by state anxiety reduced the MD values in the lenticular nucleus. *Single-headed arrows* indicate the direction of the observed regression. *Two-headed arrows* indicate a hypothetical correlation. The numbers on the *arrows* represent standardised regression coefficients. Error components are omitted for simplicity. *GSE* general self-efficacy, *MD* mean diffusivity

Figure 3 shows the link between GSE and the mean MD values within the ROI analyses mediated by individual differences in state anxiety. Among Models 1, 2, and 3, Model 2 (increasing MD values in the lenticular nucleus, with a correlation between the globus pallidum and the putamen, decreased the GSE affected by state anxiety) provided the best fit [goodness of fit (GFI) = 1.000, adjusted goodness of fit (AGFI) = 0.999, comparative fit index (CFI) = 1.000, root mean square error of approximation (RMSEA) < 0.001). The results for the other two models were as follows: GFI = 0.781, AGFI = 0.269, CFI = 0.165, RMSEA = 0.524 for Model 1; and GFI = 0.781, AGFI = 0.271, CFI = 0.172, RMSEA = 0.522 for Model 3.

## Discussion

To the best of our knowledge, the present study is the first to identify the specific direct associations between self-efficacy and brain structures in healthy individuals at the whole-brain level. Consistent with the stated hypothesis, self-efficacy scores on the GSES were associated with lower MD values in the lenticular nucleus (putamen and globus pallidum).

There are several possible mechanisms potentially underlying the relationship between GSE and the lenticular nucleus. For example, motivation is related to self-efficacy (Bandura 1977), and the motivational loop connects to the ventral striatum, including the putamen, and is sensitive to prediction error and reward uncertainty (Haruno and Kawato 2006). The putamen has been implicated primarily in motor control and learning habits and skills (Balleine and O’Doherty 2010; Durieux et al. 2011), while appropriate actions are selected by the motor corticostriatal loop, which connects motor planning areas to the putamen (Seger 2008). Interestingly, during the initial stages of skill acquisition, a reduced putamen volume is predictive of poorer performance (Raz et al. 2000), but functional connections between the sensorimotor cortex and the posterior putamen progressively strengthen as subjects learn stimulus–response tasks (Horga et al. 2015). It has also been shown that recall-related activation is related to the posterior putamen, because this type of activation is correlated with recall efficacy (Bedard and Sanes 2014). Furthermore, a reduced putamen volume is also predictive of poorer performance in a pursuit rotor task (Raz et al. 2000).

In terms of cognitive adjustments, stress facilitates dorsal striatum-dependent habit memory at the expense of hippocampus-dependent cognitive memory (van Schouwenburg et al. 2010). Similarly, the globus pallidum seems to be related to learning and adjustment. The dorsolateral posterior putamen/globus pallidum region may play a central role in the development and control of habitual behaviours (learning) in humans (Tricomi et al. 2009). Furthermore, the internal segment of the globus pallidum projects to the thalamus and brainstem nuclei that control motor behaviours (adjustment) (Hong and Hikosaka 2008). Thus, insufficient levels of function in the lenticular nucleus may lead to less self-efficacy.

There are two possibilities that may explain the present findings [i.e. cause (Models 1 and 2) and effect (Model 3)], although Models 1 and 3 did not fit well. The first possibility is that naturally lower MD (higher neuronal density) in the lenticular nucleus reflects better functioning in this region, which in turn may result in higher self-efficacy (cause). This idea is supported by previous findings showing that individual differences in GSE are due primarily to genetic factors (Waaktaar and Torgersen 2013), and thus, an inherited lower MD in the lenticular nucleus might lead to higher self-efficacy. The second possibility is that higher self-efficacy somehow influences the structure of the lenticular nucleus, such that higher self-efficacy leads to a decrease in MD (increasing neuronal density) in this region (effect). Not surprisingly, several types of interventions improve GSE compared with control groups. For example, cognitive-behavioural coping skill training increases the GSE of university students (Smith 1989; Molla Jafar et al. 2015). Moreover, interventions that include feedback regarding one’s past performance or the performance of others produce the highest levels of self-efficacy (Ashford et al. 2010). Neuronal turnover in the striatum appears to be restricted to interneurons (Ernst et al. 2014), and the vast majority of striatal neurons are medium spiny neurons characterized by high spine density (Kreitzer and Malenka 2008). Thus, striatal plasticity alters the transfer of information throughout basal ganglia circuits and may represent a key neural substrate for adaptive motor control and procedural memory (Kreitzer and Malenka 2008).

MD is a useful tool for the detection of the neural correlates of self-efficacy. In the present study, the MD analyses revealed a negative correlation between the lenticular nucleus and GSE. An explanation is warranted regarding why we only detected significant GSE-related regions using MD; the discrepancy between our previous study using rWMD (Nakagawa et al. 2015) and this study should be clarified. As we explained previously (Takeuchi et al. 2016a), changes in MD are sensitive to neural plasticity, especially in the dopaminergic system, based on cognition, such as functional adaptation. MD seems to be useful in detecting specific relationships between myelin loss, axonal damage, and diffusivity (Winston 2012). Assuming that MD reflects the density of widespread axonal terminals in the striatum, dopamine synthesis may be related to the density of dopaminergic neuronal fibres (Kawaguchi et al. 2014). Dopaminergic function in the basal ganglia is thought to be a key regulator contributing to behavioural adaptation (Nieoullon and Coquerel 2003; Schiffer et al. 2015), because the firing of dopamine neurons may provide a learning signal that guides future behaviours by modulating motivation and altering one’s willingness to initiate behaviour and expend effort (Hamid et al. 2016). Striatal dopamine may also play a role in the dynamic corticostriatal activation that occurs while encoding new motor memories during skill acquisition (Kawashima et al. 2012). Thus, because the MD analyses revealed that the neural correlates of GSE appeared to be located within the dopaminergic system, MD can be used to detect the specific regions associated with GSE.

As explained in a previous study from our research group (Nakagawa et al. 2016), a decreased MD value, which represents decreased water diffusivity on an MRI scan, is related to increased tortuosity and a decreased volume fraction of the fast diffusivity extracellular compartment. Positron emission tomography (PET) studies have revealed that the capacity for dopamine synthesis is negatively related to MD in the posterior caudate and putamen (Kawaguchi et al. 2014). Interestingly, the right caudate and putamen of mice exposed to a psychostimulant (methamphetamine) exhibited an increase in MD with no changes in FA compared with mice exposed to saline (McKenna et al. 2016). This difference seems to be based on mechanisms that differentially alter brain tissue dependent on the neural location (McKenna et al. 2016).

The present study has several limitations that should be noted. First, because this study used a cross-sectional design, the results cannot determine a causal relationship between self-efficacy and the lenticular nucleus. Prospective studies confirming the direction of causality are necessary to confirm the present findings. Second, this study included healthy young participants with high levels of education, and this may have resulted in a selection bias, because scores on the GSES are correlated with education level (Sherer et al. 1982). Finally, dopamine was not measured directly in this study, and thus, future investigations should include more sensitive measures of dopamine function, such as PET scans. Nonetheless, the present findings indicated that GSE may be associated with cellular changes in the lenticular nucleus, and that this process likely involves motivation, physical activity, learning, the willingness to initiate behaviour and expend effort, and adjustment.

## Electronic supplementary material

Below is the link to the electronic supplementary material.

Supplementary material 1 (DOC 49 KB)
