# Supplementary material for: Lenticular nucleus correlates of general self-efficacy in young adults
Source: Brain Struct Funct. 2017 Mar 28;222(7):3309–18. doi: 10.1007/s00429-017-1406-2 (PMC5585303; doi:10.1007/s00429-017-1406-2)
Supplement: Supplementary file 1 — Supplementary material 1 (DOC 49 KB) [file 429_2017_1406_MOESM1_ESM.doc]

**Supplemental Methods**

*Subjects*

All of the participants in the present study were university or post-graduate students with normal vision, no history of neurological or psychiatric illnesses, and no reports of recent psychoactive or antipsychotic drug use. Handedness was evaluated using the Edinburgh Handedness Inventory (Oldfield 1971). It should also be noted that the following descriptions of the study procedures were reproduced mostly from our previous study that used the exact same methods to investigate the same issues (Takeuchi et al. 2010b, 2015b; Nakagawa et al. 2016a). Additionally, some of the participants in these studies later became participants in subsequent interventional studies (the psychological and imaging data recorded before the intervention were used in the present study) (Takeuchi et al. 2014). The psychological tests and MRI scans not described in the present study were performed together with the tests that were described in this study.

All participants were recruited either by advertisements on bulletin boards at Tohoku University or via e-mail contact with individuals who had participated in previous experiments in our laboratory. All of the participants underwent health examinations, including eyesight assessments using an auto refractometer (Shin-Nippon ACCUREF 8001 Auto Refractometer, Ajinomoto Trading Inc.; Tokyo, Japan). Following the initial recruitment process, the participants were informed that those with diseases could not participate in this experiment, and then they were continuously notified of any other exclusion criteria after this preliminary contact. As we described previously (Nakagawa et al. 2016b), Japanese schools typically provide annual health check-ups. The medical check-up for first-year students is performed in early April, before classes start. After participants have completed the health examination, the Health Administration Centre is available to help with retesting, explaining the results, providing health-related guidance, hospital referrals, and so on. In this manner, potential participants could be excluded prior to visiting the laboratory. If, for some reason, recruited participants that fulfilled the exclusion criteria came to the laboratory, they were asked to return home. The number of excluded participants could not be verified, because some participants were dropouts, and there was a lack of records detailing the initial preliminary contacts.

***Image Acquisition***

*Acquisition of diffusion images*

Magnetic resonance (MR) images were acquired for phase correction and signal stabilization, but these were not used as reconstructed images. The mean diffusivity (MD) map has been utilized in several of our previous studies (Takeuchi et al. 2010a, 2013). Additionally, the results from the analyses that assessed these image-generated results were congruent with those of previous studies in which other methods were used (Taki et al. 2013; Barnea-Goraly et al. 2005), which suggests that this method is valid. These procedures also involved corrections for motion and distortion caused by eddy currents, and all calculations were performed using a previously proposed method (Le Bihan et al. 2001).

***Pre-processing and Analyses of Structural Data***

*Voxel-based morphometry (VBM) data*

Regional grey matter density and volume (rGMD and rGMV) were calculated. The T1-weighted images of each individual were segmented into six tissues using the default parameter settings of a segmentation algorithm in Statistical Parametric Mapping software (SPM12) implemented in Matlab (Mathworks Inc.; Natick, MA, USA). However, there were three exceptions: 1) affine regularization was performed in accordance with the Asian template, 2) the sampling distance (approximate distance between the sampled points when estimating the model parameters) was 1 mm, and 3) the thorough clean option was used to remove any odd voxels from the segmented images.

The template for the diffeomorphic anatomical registration exponentiated lie algebra (DARTEL) procedure was created using imaging data from 800 subjects (400 males and 400 females) who participated in this project, and the following methods and descriptions were reproduced from our previous study (Takeuchi et al. 2015a). The resulting images were spatially normalized to the Montreal Neurological Institute space to obtain images with 1.5 × 1.5 × 1.5 mm^3^ voxels. Next, a volume change correction (modulation) was performed by modulating each voxel with the Jacobian determinants derived from the spatial normalization, which allowed for determination of regional differences in the absolute amount of brain tissue (Ashburner and Friston 2000).

*MD data*

Using a previously validated two-step new segmentation algorithm of diffusion images and a previously validated DARTEL-based registration process (Takeuchi et al. 2013), all images, including regional grey matter segments (rGMD map), regional white matter segments (rWMD map), and the cerebrospinal fluid (CSF) segments (regional CSF density [rCSFD] map) of diffusion images, were normalized; the voxel size for all normalized MD images and segmented images was 1.5 × 1.5 × 1.5 mm^3^. For these procedures, the template for the DARTEL process that was created in our previous study, which was developed using the participants in the same project (for details, see Takeuchi et al. 2013), was utilized. Subsequently, the analyses of the MD images from the normalized images of the MD, rGMD, and rCSFD maps were used to create images in which the areas that were least likely to be grey or white matter in the averaged normalized rGMD and rWMD images (defined as gray matter tissue probability + white matter tissue probability < 0.99) were removed to exclude the strong effects of CSF on MD throughout the analyses. These images were then smoothed (6 mm full-width at half maximum [FWHM]) and carried through to the second-level analyses of MD. The following descriptions were reproduced from our previous study (Nakagawa et al. 2015).

***Statistical Group-level Analyses of Imaging and Behavioural Data***

Multimodality voxel-wise multiple regression analyses were performed to investigate the associations between MD and the General self-efficacy Scale (GSES) score.

The English in this document has been checked by at least two professional editors, both native speakers of English. For a certificate, please see:

http://www.textcheck.com/certificate/5Cp6k3

**Supplemental references**

Ashburner J, Friston KJ (2000) Voxel-based morphometry-the methods. Neuroimage 11 (6):805-821

Barnea-Goraly N, Menon V, Eckert M, Tamm L, Bammer R, Karchemskiy A, Dant CC, Reiss AL (2005) White matter development during childhood and adolescence: a cross-sectional diffusion tensor imaging study. Cereb Cortex 15 (12):1848-1854

Le Bihan D, Mangin JF, Poupon C, Clark CA, Pappata S, Molko N, Chabriat H (2001) Diffusion tensor imaging: concepts and applications. Journal of Magnetic Resonance Imaging 13 (4):534-546

Nakagawa S, Takeuchi H, Taki Y, Nouchi R, Kotozaki Y, Shinada T, Maruyama T, Sekiguchi A, Iizuka K, Yokoyama R, Yamamoto Y, Hanawa S, Araki T, Miyauchi CM, Magistro D, Sakaki K, Jeong H, Sasaki Y, Kawashima R (2016a) Basal ganglia correlates of fatigue in young adults. Scientific reports 6:21386. doi:10.1038/srep21386

Nakagawa S, Takeuchi H, Taki Y, Nouchi R, Sekiguchi A, Kotozaki Y, Miyauchi CM, Iizuka K, Yokoyama R, Shinada T, Yamamoto Y, Hanawa S, Araki T, Hashizume H, Kunitoki K, Sassa Y, Kawashima R (2015) White matter structures associated with loneliness in young adults. Scientific reports 5:17001. doi:10.1038/srep17001

Nakagawa S, Takeuchi H, Taki Y, Nouchi R, Sekiguchi A, Kotozaki Y, Miyauchi CM, Iizuka K, Yokoyama R, Shinada T, Yamamoto Y, Hanawa S, Araki T, Kunitoki K, Sassa Y, Kawashima R (2016b) Sex-related differences in the effects of sleep habits on verbal and visuospatial working memory. Frontiers in psychology 7. doi:10.3389/fpsyg.2016.01128

Oldfield RC (1971) The assessment and analysis of handedness: the Edinburgh inventory. Neuropsychologia 9 (1):97-113

Takeuchi H, Sekiguchi A, Taki Y, Yokoyama S, Yomogida Y, Komuro N, Yamanouchi T, Suzuki S, Kawashima R (2010a) Training of Working Memory Impacts Structural Connectivity. J Neurosci 30 (9):3297-3303

Takeuchi H, Taki Y, Nouchi R, Hashizume H, Sekiguchi A, Kotozaki Y, Nakagawa S, Miyauchi CM, Sassa Y, Kawashima R (2014) Effects of Multitasking-Training on Gray Matter Structure and Resting State Neural Mechanisms. Human Brain Mapping 35 (8):3646-3660. doi:10.1002/hbm.22427

Takeuchi H, Taki Y, Nouchi R, Hashizume H, Sekiguchi A, Kotozaki Y, Nakagawa S, Miyauchi CM, Sassa Y, Kawashima R (2015a) The structure of the amygdala associates with human sexual permissiveness: Evidence from voxel-based morphometry. Hum Brain Mapp 36 (2):440-448

Takeuchi H, Taki Y, Nouchi R, Sekiguchi A, Hashizume H, Sassa Y, Kotozaki Y, Miyauchi CM, Yokoyama R, Iizuka K, Seishu N, Tomomi N, Kunitoki K, Kawashima R (2015b) Degree centrality and fractional amplitude of low-frequency oscillations associated with Stroop interference. Neuroimage 119 (1):197-209

Takeuchi H, Taki Y, Sassa Y, Hashizume H, Sekiguchi A, Fukushima A, Kawashima R (2010b) Regional gray matter volume of dopaminergic system associate with creativity: evidence from voxel-based morphometry. NeuroImage 51 (2):578-585. doi:S1053-8119(10)00265-X [pii]

10.1016/j.neuroimage.2010.02.078

Takeuchi H, Taki Y, Thyreau B, Sassa Y, Hashizume H, Sekiguchi A, Nagase T, Nouchi R, Fukushima A, Kawashima R (2013) White matter structures associated with empathizing and systemizing in young adults. Neuroimage 77 (15):222-236

Taki Y, Thyreau B, Hashizume H, Sassa Y, Takeuchi H, Wu K, Kotozaki Y, Nouchi R, Asano M, Asano K (2013) Linear and curvilinear correlations of brain white matter volume, fractional anisotropy, and mean diffusivity with age using voxel-based and region of interest analyses in 246 healthy children. Hum Brain Mapp 34 (8):1842-1856
